# Visual and Auditory Hallucinations: An Uncommon Side Effect of Levetiracetam in an Elderly Patient

**DOI:** 10.7759/cureus.30668

**Published:** 2022-10-25

**Authors:** Hina Tahir, Faraz Ayyaz, Uruakanwa Ekwegh

**Affiliations:** 1 Geriatrics, Manchester Royal Infirmary/Manchester University NHS Foundation Trust, Manchester, GBR; 2 General Surgery, North Manchester General Hospital, Manchester, GBR

**Keywords:** levetiracetam, auditory visual hallucinations, levetiracetam side effect, non-convulsive seizure, subdural hemorrhage

## Abstract

This report focuses on a 77-year-old female who was admitted to an acute geriatrics unit at a tertiary care hospital with a history of recurrent falls and right leg weakness. Computed tomography of the head demonstrated acute left subdural hemorrhage with a possible extradural component at the vertex. This was causing mass effect with effacement of the left superior, frontal, and parietal lobes with no hydrocephalus or fracture. She had an episode of non-convulsive seizure secondary to a head injury and was commenced on levetiracetam. The patient started to display previously unreported visual and auditory hallucinations within four days of starting the medication. After ruling out other potential causes of the symptoms, our team narrowed it down to levetiracetam-induced hallucinations and subsequently discontinued the medication. We commenced pregabalin for seizure control, and there were no further episodes of hallucinations.

## Introduction

Levetiracetam is an anti-epileptic drug that has been in use since 2000 [1]. It was approved by the Food and Drug Administration (FDA) in the United States and by the National Institute for Health and Care Excellence (NICE) in the United Kingdom as adjunctive therapy for the treatment of focal seizures, myoclonus seizures, and primary generalized seizures. Although its mechanism of action is not fully elucidated, it has been found to target high-voltage, N-type calcium channels as well as the synaptic vesicle protein 2A (SV2A). It is rapidly absorbed and has a very high bioavailability. The pharmacokinetics are linear. It is less than 10% protein bound, and almost 66% of the dose is excreted unchanged in the urine. There is no hepatic metabolism, and because it does not inhibit or induce hepatic enzymes, there are fewer pharmacokinetic interactions and a wide therapeutic index [2]. Its common side effects in randomized adjunctive trials in adults have included headaches, somnolence, asthenia, gastrointestinal discomfort, increased risk of infection, skin reactions, vertigo, and vomiting, as reported by the British National Formulary. Hallucinations are one of the uncommon side effects of therapy with levetiracetam. Here, we report a case of levetiracetam-induced hallucinations in an elderly non-epileptic lady to highlight this uncommon side effect.

## Case presentation

A 77-year-old female presented to a tertiary care hospital with a history of a fall, having slipped off the bed while trying to mobilize. She reportedly hit her head and was unable to get up due to a combination of right leg pain and weakness. Her background was significant for recurrent falls associated with L5 nerve root impingement and a previous L3/L4 fracture. She also had cervical myelopathy and an infrarenal abdominal aortic pseudo-aneurysm. Pertinent negatives in her systemic review included the absence of syncope, chest pain, palpitations, shortness of breath, or any previous anticoagulant medications. On examination, she was normotensive, alert, and oriented, and her only neurological deficit appeared to be right leg weakness, with pain being a limiting factor in the examination.

**Figure 1:**
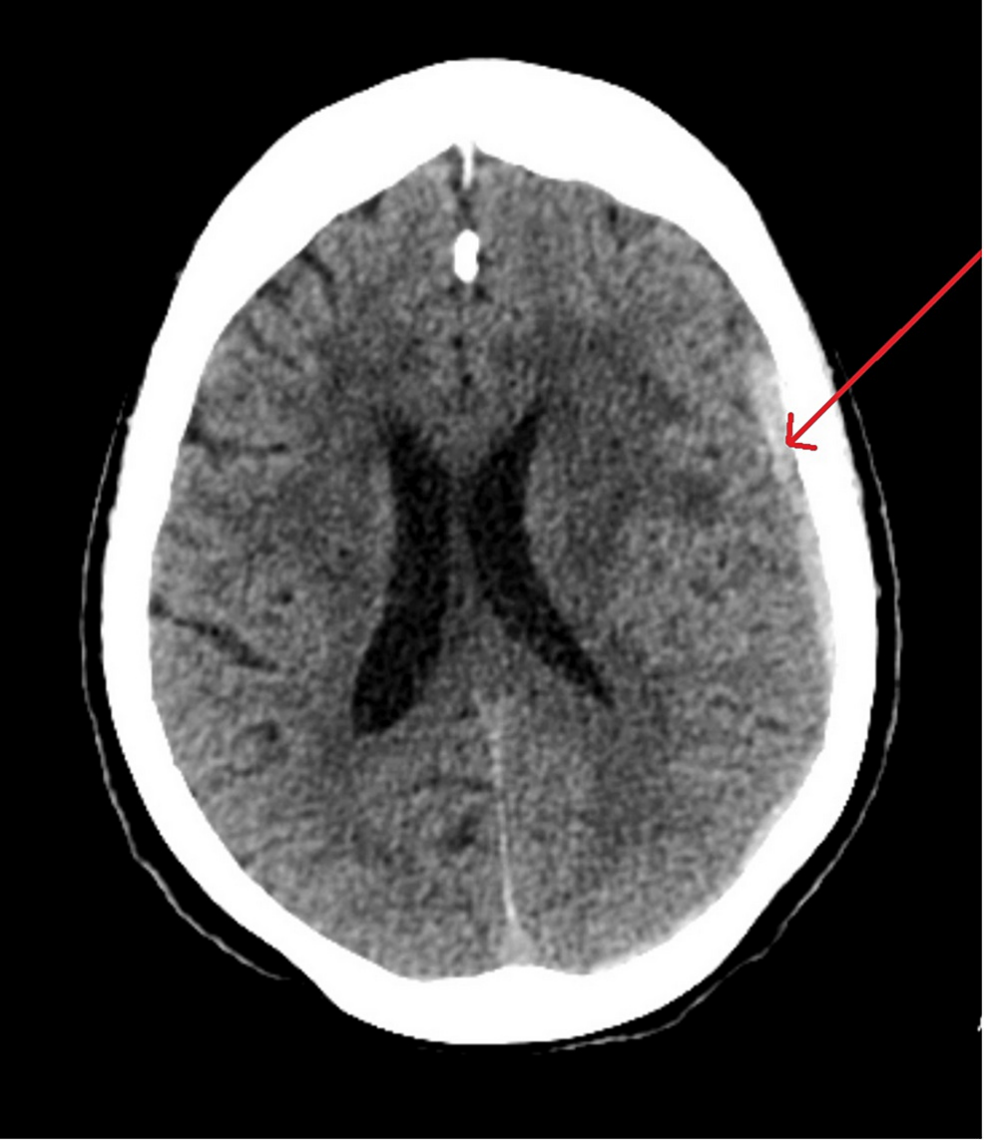
Axial view of computed tomography of the brain: acute subdural hematoma pointed by the red arrow.

She underwent computed tomography (CT) scan of her brain (due to her age, reported head injury, and possible focal neurological finding) which demonstrated an acute left subdural hemorrhage (SDH) causing mass effect with effacement of left superior frontal and parietal lobes (Figure 1).

The neurosurgical team was open to intervention but would be guided by subsequent clinical and radiological findings as there was no current indication for acute craniotomy and evacuation (high mortality and morbidity risk). Hours into her admission she was observed having an unresponsive episode for a couple of minutes while seated. We rediscussed this acute finding with the neurosurgical team on-call who determined that this was likely non-convulsive epilepsy secondary to the SDH and she was commenced on levetiracetam orally at 500 mg twice daily. She was transferred to a geriatrics ward for medical monitoring and therapy interventions.

Within four days of treatment with levetiracetam, she reported to the medical team that she was experiencing auditory and visual hallucinations. She described seeing an “elephant being killed” and could hear its sound as well. We used the Montreal Cognitive Assessment (MoCA) [3] tool to screen and the 4 A’s Test (4AT) [4] to screen for delirium. Both screening tools concluded that delirium and severe cognitive impairment were unlikely. We also repeated a confusion screen (full blood count, liver function tests, renal profile, vitamin B12 levels, folate levels, ferritin levels, and mid-stream urine sample for any possible infections). All blood and urine investigations came back within normal limits. A subsequent repeat CT scan of the brain showed a stable SDH.

Her medications were reviewed, and we decided on a trial without levetiracetam as no specific cause for the symptoms described by the patient could be singled out. She was put on a seizure chart for monitoring as per hospital protocol, and prescribed pregabalin to prevent her from developing further seizures while also improving her pain. No further hallucinations were reported by the patient after two days of stopping the drug.

**Figure 2:**
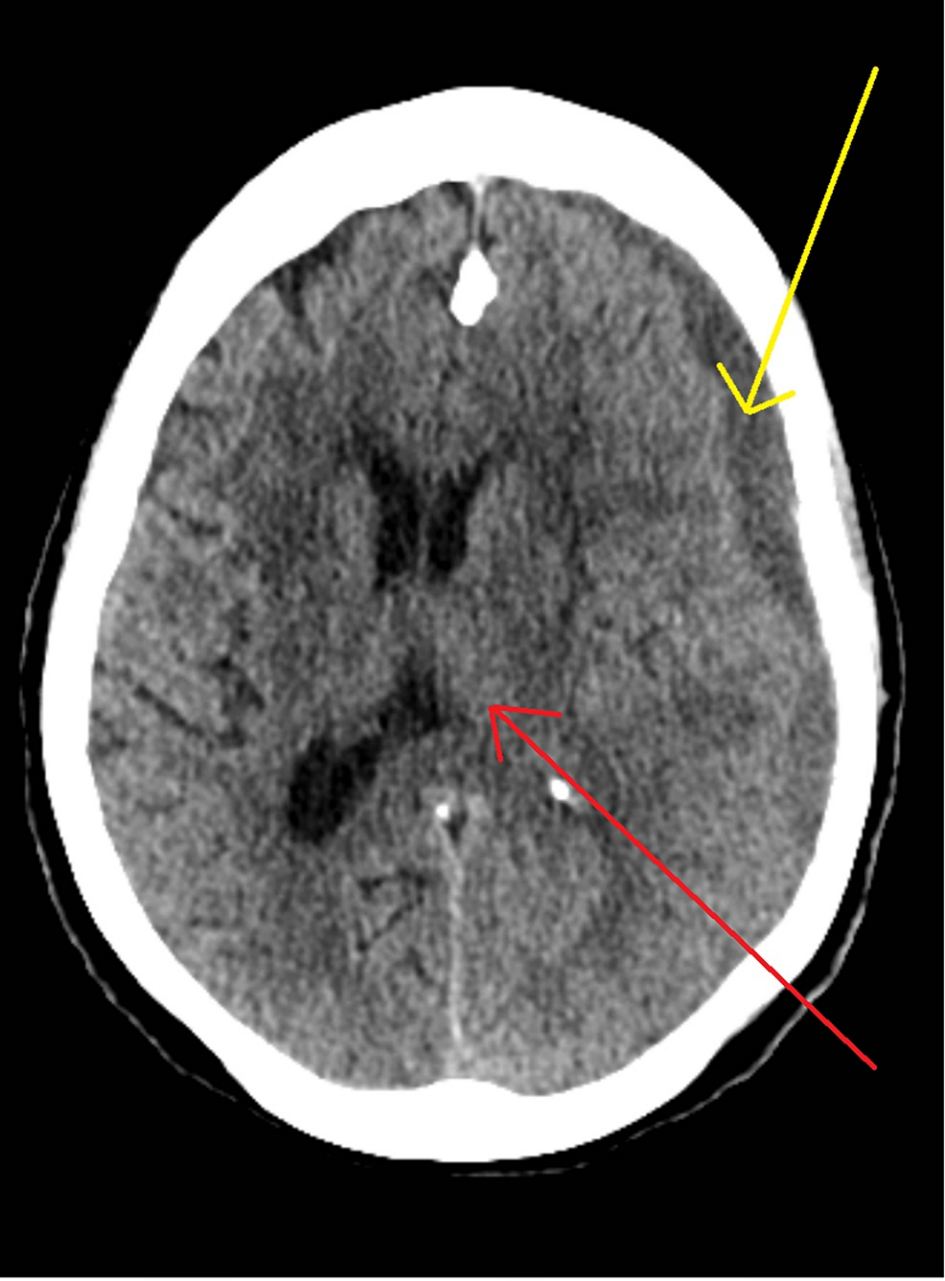
Axial view of computed tomography of the brain showing chronic subdural hematoma (yellow arrow) and herniation with midline shift (red arrow).

After consultation with the neurosurgical team, a CT of the brain was obtained two weeks after the aforementioned event which demonstrated a minimally increased mass effect with mild subfalcine herniation causing a 5 mm midline shift to right sulcal effacement, as shown in Figure 2.

She had remained cognitively stable this whole time with no reported hallucinations. As planned by the neurosurgical team on admission, the patient was transferred to the neurosurgical unit for operative intervention. The patient remained there until discharge from the hospital.

## Discussion

Levetiracetam is a piracetam analog; its mechanism of action involves the inhibition of N-type calcium channels, modulation of gamma-aminobutyric acid and glycine receptors, and binding to SVA2. Hallucinations are one of the uncommon side effects of levetiracetam [5]. In patients who have been put on levetiracetam, previous studies have reported patients developing psychiatric adverse events ranging between 13.5% and 16% [6,7].

After ruling out other causes, we suspected levetiracetam was the culprit behind the new symptoms, and it was discontinued. A subsequent resolution of hallucinations within two days of stopping the drug pointed toward the diagnosis of levetiracetam-induced hallucinations. Much as it has a good side effect profile generally, Levetiracetam can cause a wide spectrum of behavioral adverse effects. Of these, psychiatric side effects (mood disturbances, behavioral changes, cognitive changes, and hallucinations) are seen in up to 13.3% of adults [2].

Although mood and gait instability have also been reported with the use of levetiracetam in aged patients, it is considered safe and efficient for them [8]. However, these patients are also more at risk of adverse effects of medication. Therefore, as in our case, it is imperative that side effects of medication are considered in the differential diagnoses of new symptoms in elderly patients. We used the Naranjo probability scale retrospectively to assess the likelihood of our patient having developed an adverse event of levetiracetam [9]. On the scale, our patient scored 5 which falls in the “Probable” category of having developed an adverse reaction to the drug.

This case serves to highlight the potential rare side effect of levetiracetam, and the complete resolution of hallucinations after stopping the drug further supports the study.

## Conclusions

Hallucinations are an uncommon side effect of levetiracetam. This case report focuses on an elderly lady who was commenced on levetiracetam for non-convulsive epilepsy secondary to a head injury. She later developed levetiracetam-induced hallucinations, and on withdrawal of the offending agent, her symptom resolved. However, we recommend further studies on levetiracetam and its hallucinogenic side effects, especially in relation to traumatic injuries.
